# Mass spectral analysis of the multikinase inhibitor BZG and its metabolites and analysis of their binding to vascular endothelial growth factor receptor-2

**DOI:** 10.18632/oncotarget.16264

**Published:** 2017-03-16

**Authors:** Yan Lou, Wenqi Qiu, Zhe Wu, Qian Wang, Yunqing Qiu, Su Zeng

**Affiliations:** ^1^ State Key Laboratory for Diagnosis and Treatment of Infectious Disease, Collaborative Innovation Center for Diagnosis and Treatment of Infectious Diseases, Key Laboratory of Precision Diagnosis and Treatment for Hepatobiliary and Pancreatic Tumor of Zhejiang Province, The First Affiliated Hospital, Zhejiang University, Hangzhou, PR China; ^2^ Laboratory of Pharmaceutical Analysis and Drug Metabolism, Zhejiang Province Key Laboratory of Anti-Cancer Drug Research, College of Pharmaceutical Sciences, Zhejiang University, Hangzhou, PR China

**Keywords:** hepatocellular carcinoma, novel multikinase inhibitor, metabolite, VEGFR-2, eHiTS

## Abstract

We previously showed that BZG is a novel multitarget kinase inhibitor, which inhibited hepatocellular carcinoma *in vivo* and *in vitro*. In the present study, we used ultra-performance liquid chromatography coupled with quadrupole time-of-flight mass spectrometry (UPLC/Q-TOF MS) to characterize BZG and its metabolites generated *in vivo*. The probable metabolic mechanism was further confirmed by analysis of Phase I and Phase II metabolism in liver microsomes and with recombinant enzymes. In addition, the binding affinities of BZG metabolites to vascular endothelial growth factor receptor 2 (VEGFR2) were predicted using electronic high throughput screening (eHiTS). The results showed that BZG underwent phase I and phase II metabolism. We detected 11 BZG metabolites and identified hydroxylation, glucuronation, acetylation, sulfonation and degradation as the major metabolic processes *in vivo* and *in vitro*. Five of the eleven metabolites showed highly favorable eHiTS energy scores that were lower than sorafenib. Knowledge of the *in vivo* metabolic pathways of BZG and its binding affinities to VEGFR2 will be beneficial for further clinical development of BZG.

## INTRODUCTION

Hepatocellular carcinoma (HCC) is the most common primary liver cancer and the third leading cause of cancer deaths worldwide [1]. Therapeutic options for advanced HCC are limited and in spite of aggressive local treatment, recurrence is very common [2]. Currently, sorafenib remains the only established systemic therapy that increases the overall survival of unresectable HCC patients by a few months [3]. Sorafenib is a multikinase inhibitor that blocks platelet-derived growth factor (PDGF), vascular endothelial growth factor (VEGF), c-kit and fibrosarcoma signaling [4]. Sorafenib has been the most effective drug in the clinical setting [2] and newer and effective drugs are necessary to clinically combat HCC.

In our previous studies, we designed a small library of compounds that were analogous to sorafenib and screened against multiple members of the tyrosine and serine/threonine protein kinase families. BZG was one of the novel inhibitors that potently and selectively inhibits activities of kinases including VEGFR, Flt3 (Fms-like tyrosine kinase), c-kit and other kinases similar to sorafenib [5, 6]. BZG also inhibited *in vitro* proliferation of a panel of human cancer cells [7]. Moreover, our previous research indicated that BZG significantly inhibited Huh-7 cell derived tumor xenografts in Balb/c nude mice [7, 8]. Studies showed relatively high concentrations of BZG in the liver and kidney providing a pharmacokinetic (PK) basis for their effective use in hepatic and renal carcinomas [9]. However, the *in vivo* metabolism and probable drug-drug interactions of BZG are unclear.

Understanding drug metabolism is critical for druggability analysis. Drug metabolites are generally responsible for the bioactivity of the parent drug. In case of sorafenib, sorafenib N-oxide is the major pharmacologically active metabolite that shows greater potency than sorafenib against VEGFR-2 [10–12]. Therefore, we investigated the anticancer activities of the BZG metabolites in this study.

HCC is a highly vascular tumor, which proliferates through angiogenesis mediated partly by VEGF and its multiple receptors including VEGFR2. VEGFR2 (also known as KDR or FLK1) is the primary receptor mediating the angiogenic activity of VEGF in distinct signal transduction pathways and regulates endothelial cell proliferation, migration, differentiation, and tube formation [13, 14]. Since high VEGFR2 expression is associated with metastases and poor prognosis of HCC in preclinical and clinical studies, inhibition of angiogenesis is a potential therapeutic target [15].

The aim of this study was to elucidate their metabolic profiles of BZG and identify its metabolites by UPLC/Q-TOF MS method. Furthermore, we performed virtual high-throughput screening to investigate the binding affinities of BZG and its metabolites to the target receptor tyrosine kinase, VEGFR-2 using the eHiTS docking software.

## RESULTS

### UPLC/ Q-TOF MS analysis of BZG

**Figure 1:**
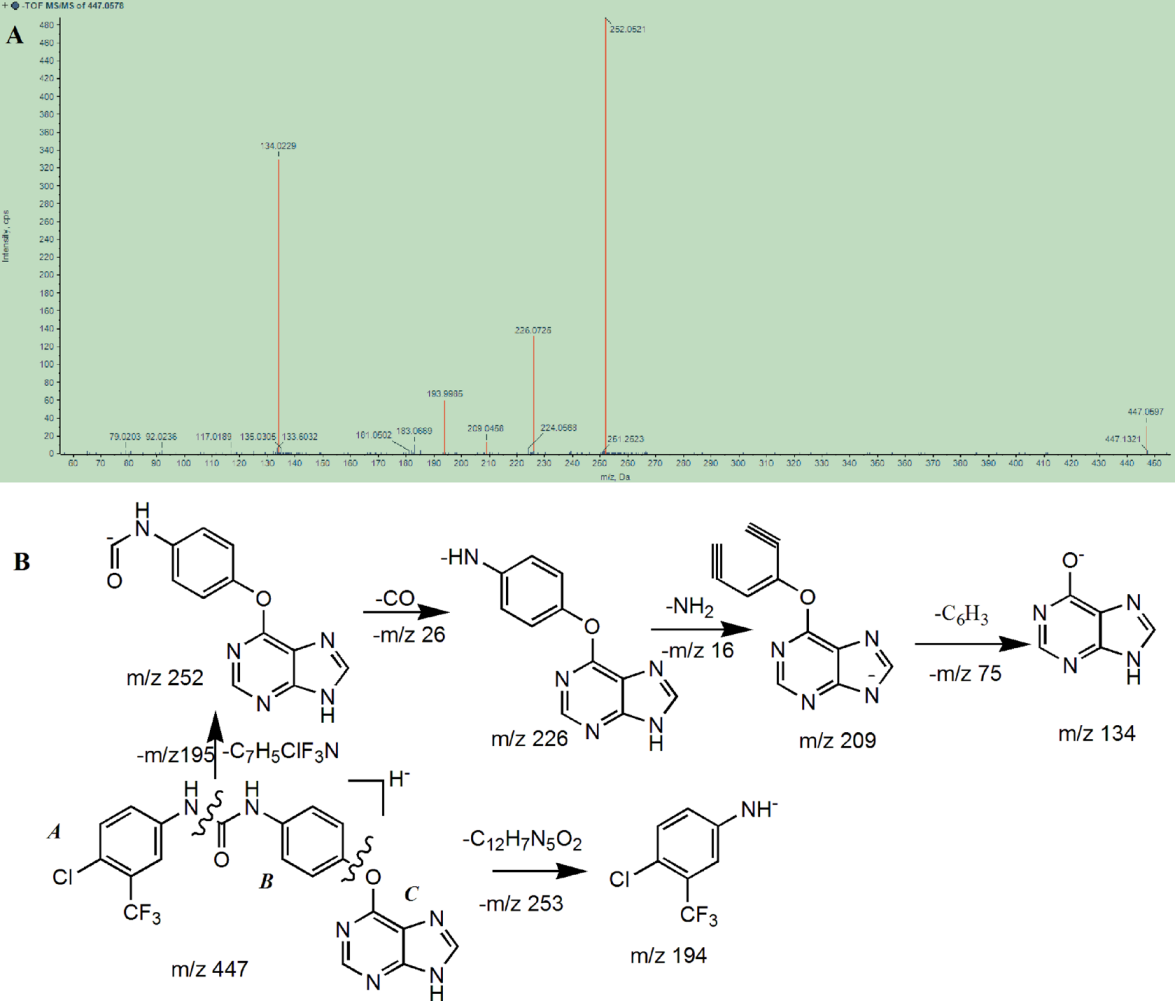
(**A**) Mass spectrum of BZG obtained on Q-TOF mass spectrometry and (**B**) Tentative structures of the most informative fragment ions for BZG.

The chromatographic and mass spectral fragmentation patterns of BZG were investigated by UPLC/Q-TOF MS (Figure 1). The protonated BZG at m/z 447 was eluted at a retention time of 12.26 min. We observed product ions at m/z 252, 226, 209, 194, and 134 (100% abundance). The fragment ions at m/z 252 and m/z 194 were generated by the cleavage of the C–N bond of the protonated molecular ion. Further loss of CO (26Da) from the fragment ion at 252 generated the fragment ion at m/z 226 and its subsequent loss of C_6_H_6_N (92Da) resulted in the fragment ion at m/z 134. Based on the results obtained, we proposed the fragmentation pathway of BZG as shown in Figure 1B. The structure of BZG was divided into parts A, B, and C (Figure 1). These fragment ions were used as references to interpret the fragment ions of the metabolites and to examine the high resolution and mass accuracy of the instrument.

### Metabolic profile of BZG

**Figure 2:**
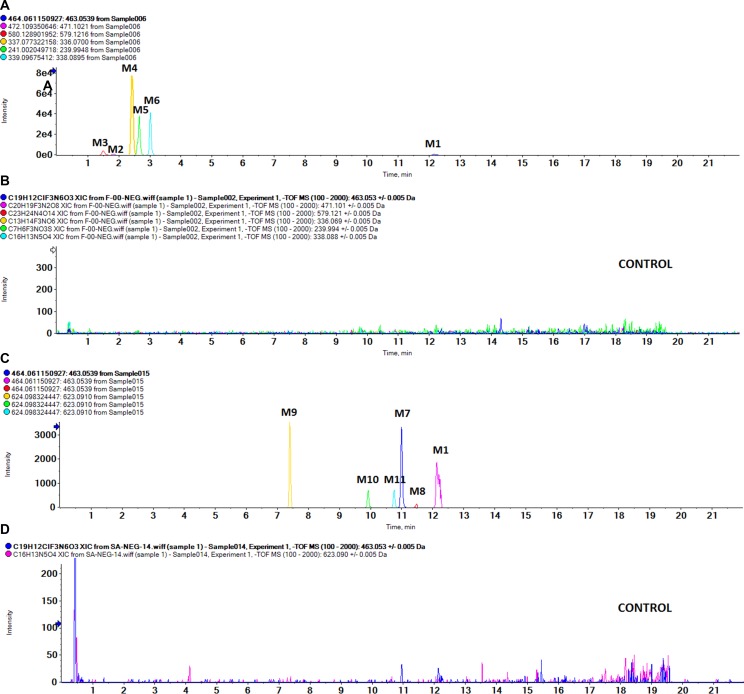
UPLC/MS chromatograms of BZG and its metabolites (**A**) *In vivo* sample; (**B**) Blank sample; (**C**) Phase I and Phase II metabolism in liver microsomes; (**D**) Control sample.

**Figure 3:**
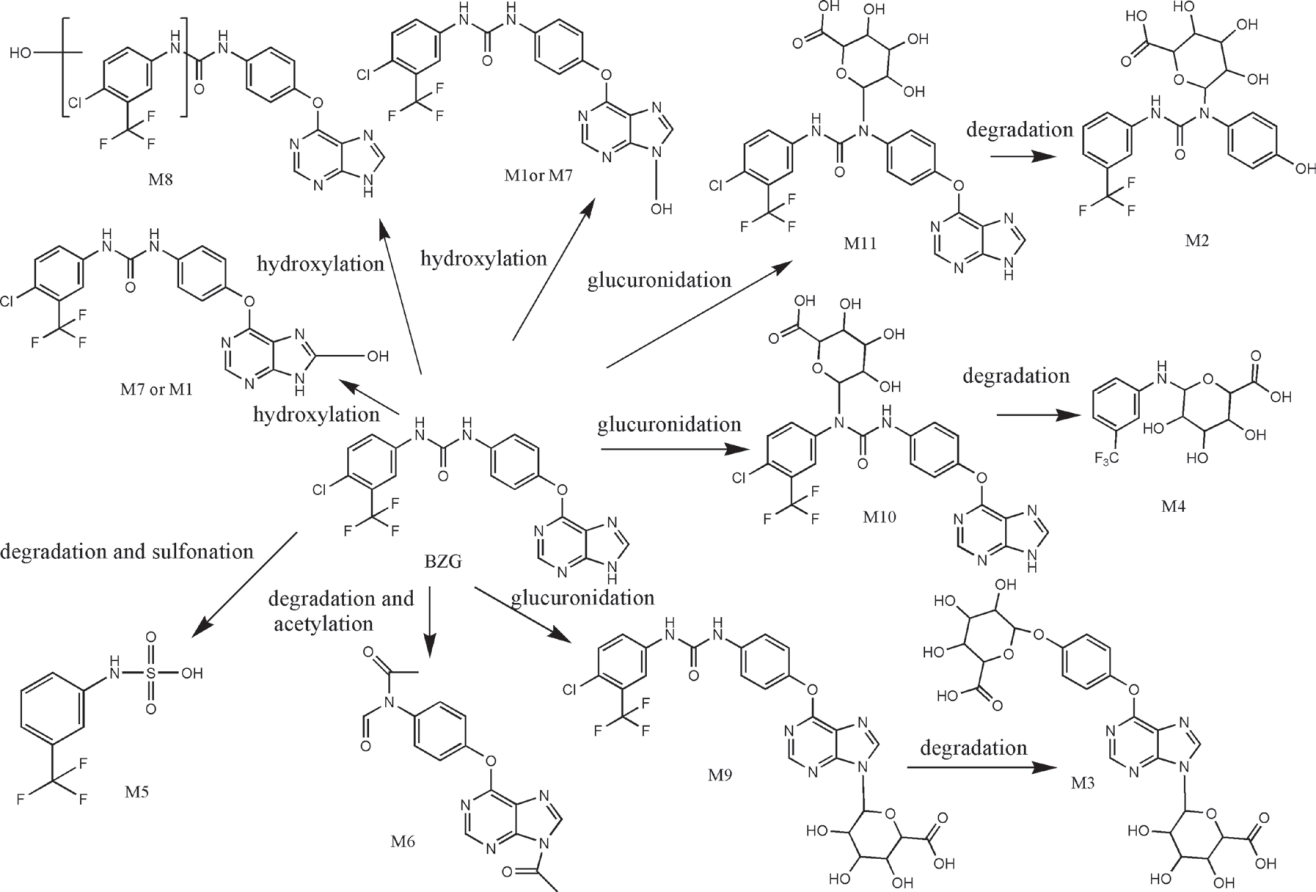
Proposed *in vivo* and *in vitro* metabolic pathways of BZG

**Figure 4:**
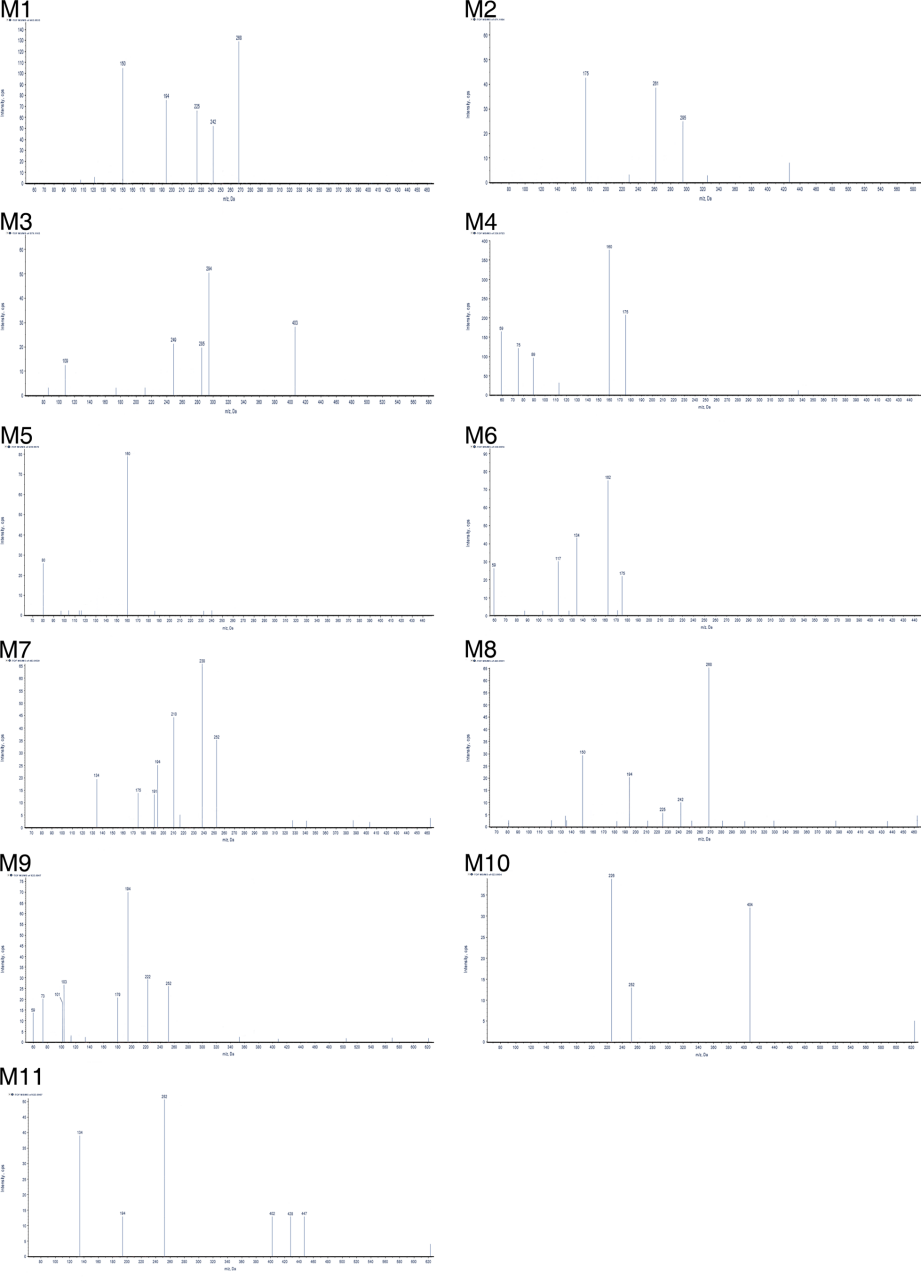
UPLC–MS/MS spectra of metabolites

As shown in Figure 2, we detected 11 metabolites of BZG *in vivo* and *in vitro*. Table 1 lists the detailed information of these metabolites, including the retention times, proposed elemental compositions, and the characteristic fragment ions. The structures of metabolites were characterized based on mass spectral fragmentation patterns. Based on the metabolites identified, we concluded that the main metabolic pathways of BZG included hydroxylation, glucuronation, acetylation, sulfonation and degradation. The representative TOF MS spectra and their proposed fragmentation pathways are shown in Figure 3 and Figure 4.

**Table 1:** Identification of BZG metabolites *in vivo* and *in vitro* using UPLC/Q-TOF MS mass spectrometry

Metabolite	Description	Retention time (min)	Formula	Measured mass [M-H]-	Calculated mass [M-H]-	Fragment ions	Found in feces	Found in bile	Found in urine	Found in plasma
M1	hydroxylation	12.16	C_19_H_12_ClF_3_N_6_O_3_	463.0533	463.0533	150, 194, 225, 242, 268	Y	Y	N	N
M7	hydroxylation	11.00	C_19_H_12_ClF_3_N_6_O_3_	463.0530	463.0533	134, 175, 191, 194, 210, 238, 252,	N	N	N	N
M8	hydroxylation	11.49	C_19_H_12_ClF_3_N_6_O_3_	463.0531	463.0533	150, 194, 225, 242, 268	N	N	N	N
M2	degradation and glucuronidation	1.85	C_20_H_19_F_3_N_2_O_8_	471.1064	471.1015	175, 261, 295	Y	Y	N	N
M3	degradation and glucuronidation	1.50	C_23_H_24_N_4_O_14_	579.1163	579.1211	109, 249, 285, 294, 403	Y	N	N	N
M4	degradation and glucuronidation	2.43	C_13_H_14_F_3_NO_6_	336.0723	336.0695	59, 75, 89, 160, 175	Y	N	N	N
M5	degradation and sulfonation	2.66	C_7_H_6_F_3_NO_3_S	239.9972	239.9942	80, 160	Y	Y	N	N
M6	degradation and acetylation	3.02	C_16_H_13_N_5_O_4_	338.0874	338.0889	59, 117, 134, 162, 175	Y	Y	N	N
M9	glucuronidation	7.40	C_25_H_20_ClF_3_N_6_O_8_	623.0907	623.0905	59, 73, 101, 103, 179, 194, 222, 252	N	N	N	N
M10	glucuronidation	9.92	C_25_H_20_ClF_3_N_6_O_8_	623.0904	623.0905	226, 252, 404	N	N	N	N
M11	glucuronidation	10.75	C_25_H_20_ClF_3_N_6_O_8_	623.0907	623.0905	134, 194, 252, 402, 428, 447	N	N	N	N

### Identification and characterization of BZG metabolites generated *in vivo*

After oral administration of BZG, 6 metabolites were obtained in the fecal samples (M1-M6) and 4 metabolites were found in bile samples (M1, M2, M5 and M6). In the urine and plasma samples, only the parent compound was detected.

### Metabolite M1 is generated by hydroxylation of BZG

The M1 metabolites were eluted at retention time of 12.16 min and showed a protonated molecular ion at m/z 463, which was 16Da higher than the protonated BZG ion at m/z 447, suggesting addition of a single oxygen atom to BZG. The major fragment ions of M1 were at m/z 268, 242, 225, 194, 150. Moreover, the fragment ions at m/z 268, 242, 225, 150 were 16Da higher than the fragment ions at m/z 252, 226, 209, 134 of the parent compound, respectively, implying that the addition of the single oxygen atom occurred in the part C. The fragment ion at m/z 194 was the same as the characteristic fragment peak of BZG, indicating that part A was intact. Based on these observations, we concluded that the M1 metabolite was generated by hydroxylation of BZG in part C.

### M2, M3 and M4 metabolites are generated by glucuronidation and degradation of BZG

The metabolite M2 eluted at a retention time of 1.82 min (Figure 2). It contained a protonated molecular ion at m/z 471, which was 24Da higher than protonated BZG ion at m/z 447. The main fragment products were at m/z 295, 261, and 175. Loss of glucuronic acid (176Da) from the protonated ion generated the major fragment ion at m/z 295, which further lost fluoride and oxygen (35Da) to form the fragment ion at m/z 261. The fragment ion at m/z 175 indicated the presence of the glucuronide residue in the structure of part B. Therefore, M2 was a monoglucuronide conjugate of BZG with removal of fluoride and part C.

M3 had a retention time of 1.50 min and was a protonated molecule at m/z 579. It had the elemental composition of C_23_H_24_N_4_O_14_, suggesting its generation by addition of C_12_H_16_O_12_ (glucuronidation) and loss of part A from BZG. The high energy mass spectrum of M3 showed fragment ions at m/z 109, 249, 285, 294, and 403. The fragment ion at m/z 403 was due to a loss of 176Da from the protonated ion, followed by the loss of C_6_H_5_O_2_ (109Da) to generate the fragment ion at m/z 294. The fragment ion at m/z 294 further lost COOH (45Da) to form the fragment ion at m/z 249. In addition, the major fragment ion at m/z 285 was formed by the cleavage of the C–O bond of the protonated ion followed by loss of C_6_H_8_O_6_ (176Da) to form the fragment ion at m/z 109. This indicated the presence of another glucuronide residue in the structure.

Metabolite M4 was eluted at a retention time of 2.43 min, showing a protonated molecular ion at m/z 336. The major fragment product at m/z 160 was due to a neutral loss of 176Da from the parent BZG ion. Moreover, the characteristic fragment peaks of glucuronic acid at m/z 59, 75, 89 and 175 further indicated that the M4 metabolite was a result of glucuronidation and degradation of BZG.

### M5 metabolite is generated by sulfonation and degradation of BZG

The metabolite M5 showed an UPLC elution profile with a retention time at 2.66 min. The protonated molecular ion at m/z 240 was a degradation product since it was 231Da lower than BZG. The major fragment ions of M5 were m/z 80 and 160. The fragment ion at m/z 80 was characteristic of sulfonic acid. The fragment ion at m/z160 was formed by the loss of SO_3_H (80Da) from the precursor ion at m/z 240 that originated from a modified NH portion. Therefore, we concluded that M5 was generated by sulfonation of BZG and removal of parts B and C.

### M6 metabolite is generated by acetylation and degradation of BZG

Metabolite M6 had a retention time of 3.02 min. It had a protonated molecular weight of 338Da, which was 109Da lower than that of protonated BZG. The major fragment ions of M6 were at m/z 175, 162, 134, 117 and 59. The fragment ions at m/z 175 and 162 were generated by the cleavage of the C–O bond of the protonated molecular ion at m/z 338. The fragment ion of m/z 175 was formed by the loss of C_2_HO (41Da) from characteristic fragment ion of BZG (m/z 134), revealing acetylation on part C. Then, this fragment ion lost CH_2_CO and oxygen atom to form the fragment ion at m/z 117. The fragment ion at m/z 162 lost a CO to form the ion at m/z 134, which further lost the benzene ring to form the fragment ion at m/z 59, indicating another acetylation event. Therefore, M6 was degradation result of acetylation in parts B and C of BZG and further degradation.

### Identification of *in vitro* BZG metabolites

#### Metabolism of BZG in human liver microsomes (HLMs)

Compared with the control sample, 3 oxidative metabolites (M1, M7, and M8) were obtained in Phase I metabolism of BZG. In addition, 3 monoglucuronide conjugates of BZG (M9–M11) were detected in Phase II metabolism of BZG.

### M7 and M8 metabolites are generated by hydroxylation of BZG

Metabolites M7 and M8 were eluted at retention times of 11.00 and 11.49 min, respectively. Both showed a protonated molecular ion at m/z 463, which was 16Da higher than that at m/z 447 suggesting addition of a single oxygen atom. The major fragmentation of M7 was at m/z 210, which was 16Da higher than the fragment ion at m/z 194 of the parent BZG, implying that the modification was in part C. This fragment ion further lost either a fluorine (19Da) or a chlorine atom (36Da) to form fragment ions at m/z 191 and 175, respectively. The fragment ion at m/z 238 was generated by the addition of CO_2_ (44Da) to the ion at m/z 194. Moreover, the fragment ions at m/z 252 and 134 indicated that parts B and C were intact. The metabolite M8 had similar fragment ions as M1, suggesting that the two metabolites were isomers. Based on these observations, we concluded that M7 and M8 were generated by hydroxylation of BZG in parts A and C, respectively. However, the exact sites of hydroxylation could not be characterized.

### M9, M10 and M11 metabolites are generated by glucuronidation of BZG

The BZG metabolites M9, M10 and M11 were eluted at retention times of 7.40, 9.92 and 10.75 min, respectively. All the three metabolites showed a protonated molecular ion at m/z 623. The elemental composition of this metabolite was C_25_H_20_ClF_3_N_6_O_8_, corresponding to the monoglucuronide conjugate of BZG.

The fragment ions of M9 were observed at m/z 59, 73, 101, 103, 179, 194, 222, and 252. The fragment ions at m/z 252 and 194 were the same as those of BZG. The fragment ion at m/z 179 was generated by the loss of NH (15Da) from the characteristic ion (m/z 194). The fragment ion at m/z 222 resulted from the addition of CO (28Da) from the fragment ion at m/z 194. The fragment ions at m/z 59, 73, 101, and 103 were formed by the cleavage of the C-C or C-O bond from the glucuronic acid ion at m/z 179. Therefore, M9 were identified as monoglucuronide conjugate of BZG.

The main fragment products of M10 were at m/z 252 and 226, identical to the fragment ions of BZG. The fragment ion at m/z 404 was generated by the addition of glucuronic acid (176Da) and subsequent loss of one chlorine atom (36Da), CF_3_ (69Da) and C_5_H_3_N_4_ (119Da) from BZG. On the basis of the above analysis, M10 was identified as monoglucuronide conjugate of BZG with the site of glucuronidation on part A.

The fragment ion at m/z 447 of M11 was due to a neutral loss of 176 Da. The major fragment ions at m/z 134, 194, and 252 were the same as that of BZG. In addition, the fragment ion at m/z 428 was formed by the addition of glucuronic acid (176Da) to the characteristic fragment ion at m/z 252. Further, loss of CO (28Da) from the fragment ion at m/z 428 generated the fragment ion at m/z 402. Thus, the site of glucuronidation in M11 was on part B.

### Metabolism of BZG in human recombinant enzymes

A screening of the Cytochrome P450 (CYP) enzymes confirmed that hCYP1A2, 2B6, 2C19, and 2C8 exhibited hydroxylation activity (Table 2). Furthermore, hCYP2B6 exhibited relatively higher metabolic capacity than other CYPs. Further, experiments with recombinant human UDP- glucuronosyltransferases (hUGTs) indicated that UGT1A9 played a major role in the glucuronidation of BZG in HLMs (Table 2).

**Table 2:** Metabolites of BZG in recombinant human cytochrome P450 enzymes and UDP-glucuronosyltransferase enzymes

			recombinant human cytochrome P450 enzymes					
	CYP1A2	CYP2B6	CYP2C8	CYP2C9	CYP2C19	CYP2D6	CYP2E1	CYP3A4
Metabolites	M1	M1	M1	ND	M1	ND	ND	M7
			**recombinant human UDP-glucuronosyltransferase enzymes**					
	UGT1A1	UGT1A3	UGT1A6	UGT1A7	UGT1A9	UGT2B7	UGT2B15	
Metabolites	ND	ND	ND	ND	M9	ND	ND	

Note: ND: No detection.

### Virtual screening

**Figure 5:**
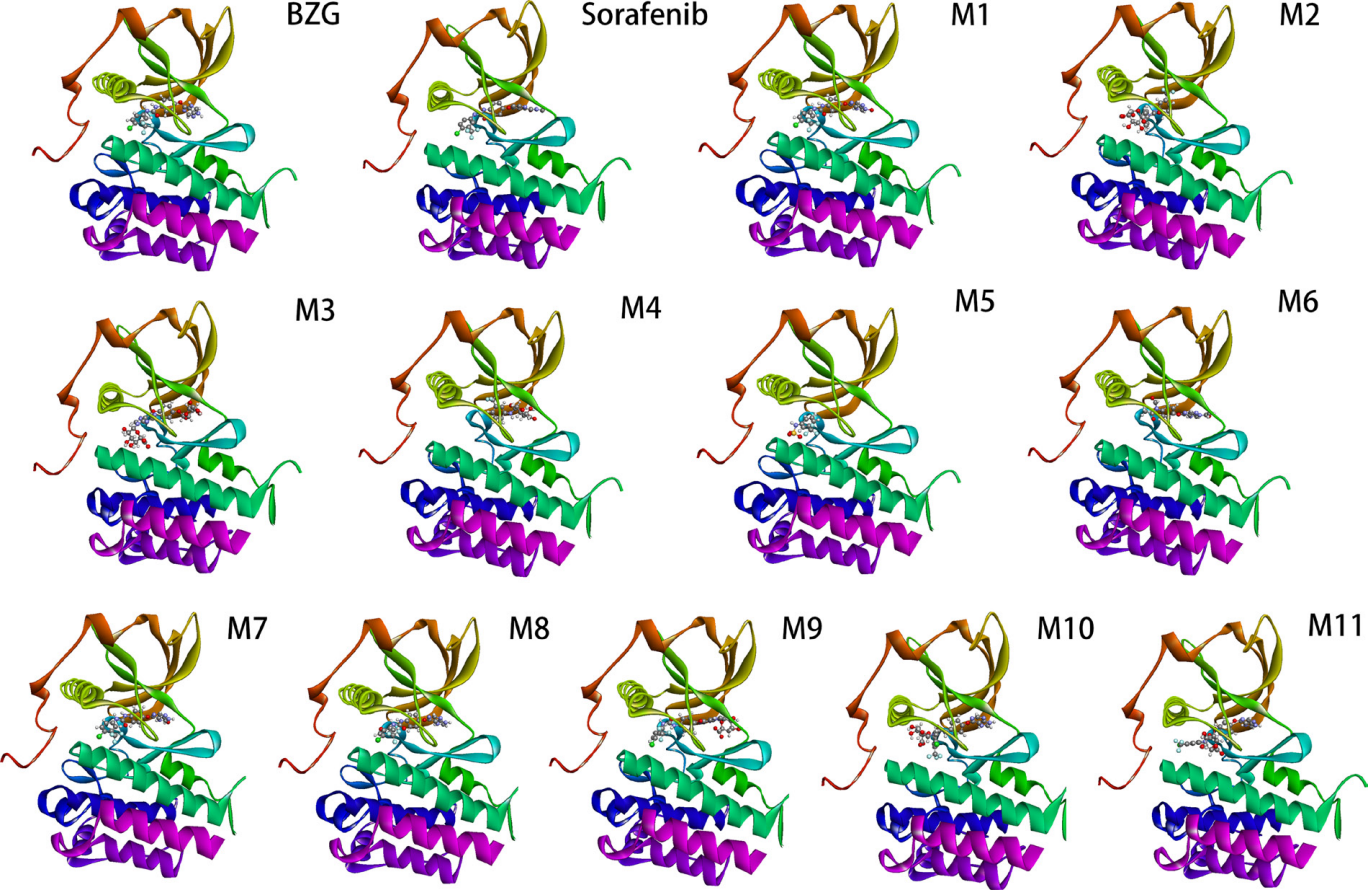
The eHiTS predicted binding model of BZG and its metabolites to VEGFR-2 active site

Based on eHiTS analyses, the tested compounds potently inhibited VEGFR-2 (Figure 5). The dock scores of the metabolites and BZG are shown in the Table 3. Five metabolites M2-3, and M9-11 showed favourable eHiTS energy scores. The eHiTS energy scores of M3 (−9.41), M11 (−7.66), M10 (−7.19), M2 (−7.03) and M9 (−6.86) were lower than sorafenib (−6.66). The M3 metabolite was the most suitable for further experiments because of its better ligand efficiency.

**Table 3:** eHiTS energy scores of BZG and its metabolites for ligand binding affinities with VEGFR-2 (Sorafenib was used as a positive control)

Name	eHiTS-Score
BZG	−3.711
M1	−3.471
M2	−7.034
M3	−9.412
M4	−5.459
M5	−5.005
M6	−6.272
M7	−3.507
M8	−3.466
M9	−6.861
M10	−7.19
M11	−7.658
sorafenib	−6.655

## DISCUSSION

An integral part of drug discovery and development is the identification of metabolites of drug formed during phase I and phase II metabolic reactions. These metabolites may either demonstrate intrinsic pharmacological activity or display specific toxicity. Previous *in vitro* and *in vivo* studies showed that sorafenib underwent oxidative metabolism by CYP3A4 and glucuronidation by UGT1A9 into 8 metabolites [16]. In this study, BZG underwent metabolism in phase I that included monohydroxylation (M7, M8) and N-hydroxylation (M1). This indicated that during phase I, hydroxylation of BZG was the major metabolic event in rats. Successive metabolic processes, including sulfonation and degradation (M5), glucuronidation and degradation (M2-4), and acetylation and degradation (M6) were also detected. M4-M6 metabolites were more abundant than others indicating that glucuronidation and degradation were the major metabolic reactions for BZG in rats. The proposed metabolic pathways of BZG are depicted in Figure 3. BZG and its degradation metabolites predominantly passed in the feces, whereas a portion of BZG metabolites were broken down by bacteria flora in the intestine. The intestinal tract plays an important role in the metabolic disposition of BZG and studies are necessary to investigate the intestinal bacteria involved in this process. BZG and its metabolites were excreted more through feces than urine. Therefore, renal elimination is not a major player in BZG clearance.

Furthermore, HLMs and recombinant enzymes are useful *in vitro* tools for predicting drug metabolic pathways and potential clinical drug-drug interactions. Our experiments with human liver microsomes and recombinant enzymes indicated that BZG underwent oxidative metabolism by hCYP1A2, 2B6, 2C19, and 2C8 (M1) and hCYP3A4 (M7) and glucuronidation by hUGT1A9 to M9. BZG had three potential glucuronidation sites and generated different products in association with UGTs. Our data also indicated that hUGT1A9 was the most efficient enzyme among all UGTs. In humans, hUGT1A9 is predominantly expressed in the liver [17]. Since many drugs are either inhibitors or inducers of hUGT1A9 [18], drug-drug interactions may occur through this pathway. There efficacy of the newly identified phase I and II metabolites needs to be further investigated.

UPLC/QTOF MS has become the cornerstone in drug metabolite identification because of its sensitivity and ability to analyze complex mixtures [19, 20]. In addition, the metabolitepilot^TM^ 1.5 software combined with neutral loss filtration and mass defect filtration technique helps identify multiple bioactive metabolites *in vivo* with short data interpretation time and high quality structural information. The results provided helpful chemical information to investigate further mechanistic aspects regarding the pharmacological role of BZG.

VEGF family members are the major growth factors that regulate HCC progression [21–23]. Preliminary data has shown that tyrosine kinase inhibitors of VEGFR2 such as sunitinib, sorafenib, and foretinib have high efficacy in HCC patients [15]. In this study, we described the computational model of the binding of BZG and its metabolites with the target protein VEGFR2 using docking methods. eHiTS has a novel flexible ligand docking method that exhaustively generates conformations and avoids severe steric clashes between receptor and ligand. Since it is a deterministic system, the results are scientifically reproducible [24]. Our studies showed that metabolites M2-3, and M9-11 efficiently inhibited the binding of VEGFR2. Further *in vivo* and *in vitro* are needed to follow-up on our findings.

In conclusion, 11 metabolites (M1–M11) of BZG were identified during phase I and phase II metabolic processes. Of these metabolites, M2-3, and M9-11 efficiently inhibited the binding of VEGFR-2. Our results suggested that the BZG and its metabolites were effective antagonists of the VEGF/VEGFR2-stimulated angiogenesis and potential candidates for further optimization. Our results provided deeper understanding regarding the metabolism of BZG and useful information regarding the safety and efficacy of BZG.

## MATERIALS AND METHODS

### Reagents

Trisodium isocitric acid, isocitric dehydrogenase, *β*-NADP and its reduced form (*β*-NADPH), UDP-glucuronic acid (UDPGA), and *β*-glucuronidase were purchased from Sigma-Aldrich (St. Louis, MO, USA). *S*-Adenosyl-*L*-methionine *p*-toluene sulfonate salt was obtained from Aladdin Reagent (Shanghai, China; purity > 80%). Calcium chloride, epsom salt, dipotassium hydrogen phosphate, potassium dihydrogen phosphate, sodium bicarbonate and sodium chloride were supplied by Sinopharm Chemical Reagent (Beijing, China). Tryptone, yeast extract, and cysteine hydrochlorate were obtained from BBI (Canada). All other chemicals were from standard commercial sources and were of the highest quality.

### Generation of *in vivo* BZG metabolites

BZG was synthesized by Shanghai Medicilon Inc. (Shanghai, China). BZG solution was prepared by dissolving the drug in a mixture of dimethyl sulfoxide:PEG 400:physiological saline in a 1:6:13 ratio by volume. Briefly, the drug was weighed and vortex mixed in a 1.5 ml tube with DMSO until the BZG dissolved followed by addition of PEG 400 and physiological. The solution was clear and no particulate matter was observed.

For *in vivo* experiments, male Sprague-Dawley rats (200–220 g) were obtained from the Animal Center of Zhejiang Academy of Medical Sciences (Hangzhou, China) and bred in a breeding room with temperature at 25°C, 50 ± 10% humidity, and a 12 h dark–light cycle. They had free access to water and rodent chow all the time. All the experiment animals were acclimatized in the above conditions for one week and fasted overnight before the experiments. The study was approved by the Animal Ethics Committee of Zhejiang University.

Then, twelve rats that were divided into two random groups were treated with 20mg·kg^−1^ BZG by oral gavage after an overnight fasting period. After administration, the urine and feces samples were collected from the metabolic cage from one group and bile samples were collected via the bile duct catheter from another group for 24 h. Rats were then euthanized with CO_2_ after completion of the studies. All samples were stored at −80°C until further analysis.

### Preparation of blood, urine, fecal and bile samples for UPLC/Q-TOF/MS analysis

Blood samples (300 μL) were collected into heparinized tubes by scissoring rat tails at 0 (predose), 0.5, 3, 8, 12, and 48 h after administration orally. After plasma collection, each blood sample was immediately centrifuged at 4000 × g for 5 min at 4°C, and the samples were stored at −20°C until analysis. Before analysis, the plasma samples were thawed to room temperature and 100 μL was vortexed with 200 μL acetonitrile in a 1.5 mL centrifuge tube for 1 min. Subsequently, the mixture was centrifuged at 13,000 × g for 20 min at 4°C. The protein-free supernatant was separated and a volume of 100 μL was pipetted into another 1.5 mL centrifuge tube and used for analysis.

A mixture of 1ml urine sample and 1ml acetonitrile was mixed by vortexing for 5min after filtration. The supernatant was transferred into another test tube and evaporated to dryness under vacuum at room temperature. Finally, the residue was reconstituted in 100 μL incipient mobile phase by vortexing for 4 min and further centrifuged at 13,000 rpm for 20 min. The supernatant was used for analysis.

The fecal samples were twice extracted ultrasonically with 3 ml·g^−1^ methanol for 30 min. The combined methanol extracts were concentrated to nearly 1.0ml under vacuum and centrifuged at 13,000 rpm for 20 min. The supernatant was stored at −80°C until analysis. The bile sample was prepared similar to the urine samples except that the extraction solution was 1:1 methanol:H_2_O by volume.

### Generation of *in vitro* metabolites

#### Phase I metabolism of BZG in human liver microsomes

Human liver microsomes were purchased from the Research Institute for Liver Diseases (Shanghai) Co. Ltd. For optimization of the incubation conditions, the linearity of metabolite formation with time (10–120 min) and protein (0.2–2 mg·ml^−1^) in HLMs was evaluated in advance. All incubation mixtures in a 100 μL total volume containing 0.1 M Tris-HCl (pH 7.4), 15 mM MgCl_2_, the NADPH-generating system, human liver microsomes (0.5 mg·ml^−1^), and 100 μM BZG were preincubated at 37°C for 3 min. The reaction was initiated by adding NADP and NADPH at 37°C and stopped after 30 min by the addition of 300 μL of ice-cold acetonitrile. After centrifugation at 13000 rpm for 10 min), the supernatant was used for analysis. Control incubations with inactive HLMs or without cofactors were also conducted in parallel.

### Phase II metabolism of BZG in human liver microsomes

Glucuronidation was investigated by incubating HLMs (0.5 mg·ml^−1^) at 37°C for 120 min in a 100 μl medium containing 0.1 M K_2_HPO_4_ buffer, 25 μg·ml^−1^ alamethicin, 10 mM MgCl_2_, 50 mM Tris-HCl (pH 7.4), and 100 mM BZG. Then 20 μL of 4.5 mM UDPGA solution was added to the reaction mixture. The reaction was terminated by the addition of 300 μL of ice cold methanol. After centrifugation, the supernatant was used for analysis. Controls with inactive HLMs or without cofactors were conducted in parallel.

The mixed incubation of oxidative and glucuronidation metabolism was investigated in 100 μL of buffer containing 0.1 M Tris-HCl (pH 7.4), 15 mM MgCl_2_, 12 mM dl-isocitrate trisodium, 0.08 unit of isocitrate dehydrogenase, 112.5 μg/ml Triton X-100, 1mg·ml^−1^ human liver microsomes, and approximately 200 μM BZG in DMSO (final concentration of DMSO did not exceed 1%). The mixture was preincubated at 37°C for 3 min. Then 2 μL of NADP/NADPH solution and 4 μL of UDPGA solution were added to the reaction mixture, and incubation was performed at 37°C for 30 min. The reaction was stopped by addition of 300 μL of ice cold methanol. After centrifugation, the supernatant was analyzed by UPLC/QTOF MS.

### Generation of *in vitro* metabolites of BZG with recombinant enzymes

Recombinant human CYP supersomes (CYP1A2, 2B6, 2C8, 2C9, 2C19, 2D6, 2E1 and 3A4) and UGT supersomes (UGT1A1, 1A3, 1A6, 1A7, 1A9, 2B7, and 2B15) expressed in baculovirus-infected insect cells were purchased from BD Biosciences. Experiments to determine *in vitro* metabolism of BZG with recombinant enzymes was conducted according to the manufacturer’s instructions. Briefly, eight human CYP supersomes (CYP1A2, 2B6, 2C8, 2C9, 2C19, 2D6, 2E1, and 3A4) and seven commercially available human UGT supersomes (UGT1A1, 1A3, 1A6, 1A7, 1A9, 2B7, and 2B15) were evaluated for their ability to metabolize 100 μM BZG. The incubation conditions were similar to those used with HLMs except that the CYP content was 50 pmol CYPmL^−1^ and the UGT concentration was 0.5 mg proteinmL^−1^.

### Virtual screening of BZG, its metabolites and their docking with VEGFR-2

The virtual screening of protein-ligand interactions of BZG and its metabolites (Sorafenib was used as a positive control) with VEGFR-2 as the target protein was performed using eHiTS 14.0 from SimBioSys Inc. While eHiTS (http://www.simbiosys.com/ehits) [24, 25] was used for the active site detection and docking, Open Babel (http://openbabel.org) was used for manipulating the ligands chemical formats and acquiring the ligands 3D structures. The scoring was according to the eHiTS-Score that is included in the eHiTS software package. The crystal structure of VEGFR-2 in complex with sorafenib (PDB code: 1AH3; http://www.rcsb.org/pdb/) was selected for molecular docking with BZG and its metabolites. However, no special preparation of the 3D structures was applied since eHiTS automatically evaluates all the possible protonation states for the ligands and enzymes. Active site detection was carried out using the ’complex’ parameter. The eHiTS program automatically detected the ligand in the complex and selected the part of target protein within a 7 Å margin around the ligand to be the active site. The compound was then docked into the active site using the highest accuracy mode of docking (accuracy parameter was set to 6).

### UPLC and MS conditions

The AB Triple TOF 5600^plus^ System (AB SCIEX, Framingham, USA) was connected to the UPLC system (Waters, Milford, MA, USA) via an electrospray ionization (ESI) interface. Chromatography was performed on the UPLC with a conditioned autosampler at 4°C using the Acquity BEH C_18_ column (50 mm × 2.1 μm., 1.7 μm particle size;Waters, Milford, MA, USA). The mobile phases were 0.1% formic acid:5 mM ammonium formate:water (A) and 0.1% formic acid:5 mM ammonium formate:acetonitrile (B). The linear gradient elution of 0–1 min, 5% B; 1–12 min, 40% B; 12–16 min, 40–70% B;16–18 min, 70–95% B; 18–19 min, 95% B was used to equilibrate the column. The flow rate was set to 0.4 mL·min^−1^ and the injection volume was 5 μL. The optimal MS conditions were: negative ion mode; source voltage was -4.5 kV; the source temperature was 550^°^C; the pressures of gas1 (Air) and gas2 (Air) were set to 50 psi; the pressure of curtain Gas (N_2_) was set to 35 psi; maximum allowed error was ± 5 ppm; declustering potential (DP) was 100V; collision energy (CE) was 10V. For MS/MS acquisition mode, the parameters were almost the same except that the collision energy (CE) was set at 40 ± 20 V, ion release delay (IRD) was set at 67 and ion release width (IRW) was set at 25.

For the 8 most intense metabolite ions, the IDA based auto MS^2^ was performed in a full cycle scan of 1s). The scan range of m/z for the precursor and product ions was set at 100–2000 Da and 50–1500 Da, respectively. The exact mass calibration was performed by the automated calibration delivery system, automatically before each analysis.

HPLC grade acetonitrile, methanol and formic acid were purchased from TEDIA Inc. (Fairfield, USA). Ultra-pure water (18.2 MΩ) was obtained from an ELGA-Pure lab Ultra system (High Wycombe, UK).

### Data processing

Mass spectral data processing of extracted ion chromatograms and calculation of the elemental compositions using potential metabolite ions was carried out using the PeakView 2.0 software. MetabolitePilot^TM^1.5 software was used to automatically identify metabolites by comparing the sample with the controls. Binding affinities were predicted by the docking program eHiTS and a scoring function for the calculation of ligand binding affinities.

## Abbreviations

UPLC/Q-TOFMS: ultra-performance liquid chromatography coupled with quadrupole time-of-flight mass spectrometry
eHiTS: electronic high-throughput screening
HCC: Hepatocellular carcinoma
VEGFR: Vascular endothelial growth factor receptors
PDGF: platelet-derived growth factor
bFGF: basic fibroblast growth factor
